# Supplementary material for: Photoactivatable Cre recombinase 3.0 for in vivo mouse applications
Source: Nat Commun. 2020 May 1;11:2141. doi: 10.1038/s41467-020-16030-0 (PMC7195411; doi:10.1038/s41467-020-16030-0)
Supplement: Supplementary file 1 — Supplementary Information [file 41467_2020_16030_MOESM1_ESM.pdf]

Supplementary Information

Photoactivatable Cre recombinase 3.0 for *in vivo* mouse applications

Morikawa *et al.*

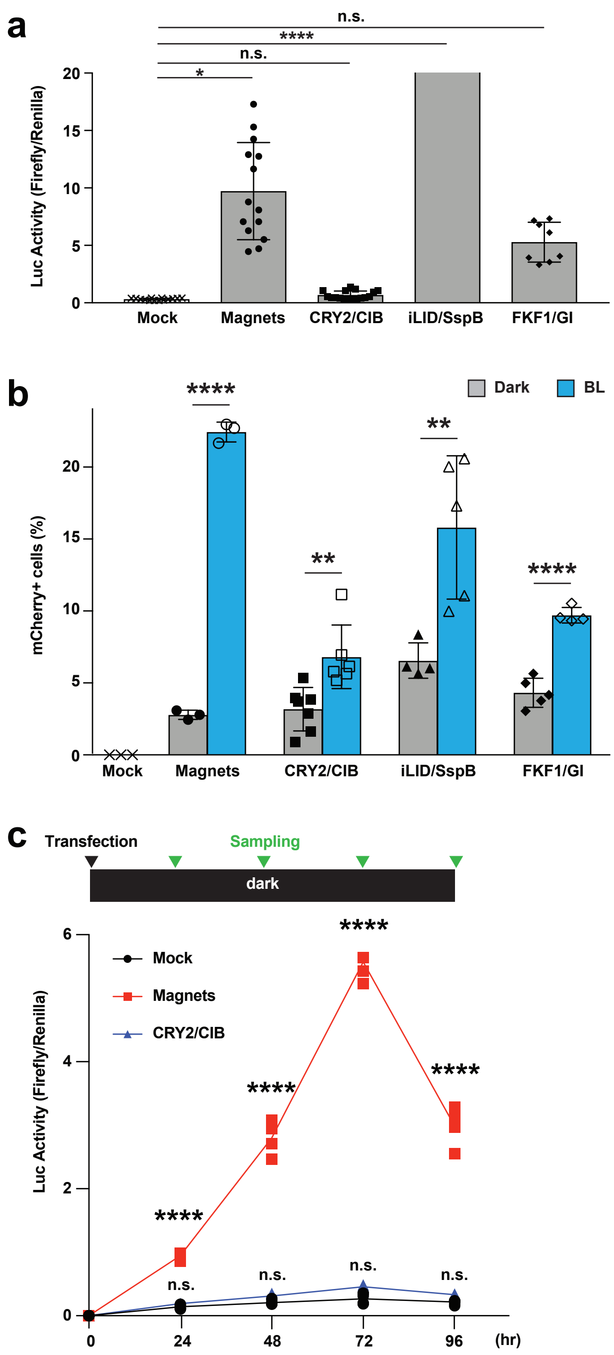

Supplementary Fig. 1 Comparison of multiple light-activated dimerizers in photoactivatable Cre dark leakiness

Supplementary Fig. 1 Comparison of multiple light-activated dimerizers in photoactivatable Cre dark leakiness.

a, Comparison of PA-Cre leak activity in the dark. Luc assays were conducted along with double-floxed inverted Fluc reporter in HEK 293T cells (same dataset shown in Fig. 1b). The transfected cells were kept in the dark for 48 h. (n.s. not significant, **P*<0.05, *****P*<0.0001; one-way ANOVA, Biologically independent samples; Mock, Magnets, iLID/SspB: *n*=14, CRY2/CIB *n*=17, FKF1/GI *n*=8, mean±s.d.). iLID/SspB dark leakiness reached to over 50 as shown in Fig. 1b.

b, Quantification of mCherry-positive cells. with blue light illumination (BL) and in dark (***P*<0.01, *****P*<0.0001; BL v.s. dark using Two-tailed *t*-test, Biologically independent samples; Mock, Magnets: *n*=3, CRY2/CIB Dark *n*=7, CRY2/CIB BL *n*=6, iLID/SspB Dark *n*=4, iLID/SspB BL *n*=5, FKF1/GI Dark *n*=5, FKF1/GI BL *n*=4, mean±s.d.).

c, Luc assays were performed in HEK 293T cells transfected with CAG promoter-mediated Magnets- or CRY2/CIB-based PA-Cre, double-floxed inverted Fluc and *Renilla* Luc control plasmids. Upper diagram shows the experimental time course. Transfected cells were maintained in dark and the cell lysate collected for Luc assay every 24 h (n.s. not significant, *****P*<0.0001; one-way ANOVA, biologically independent samples; Mock *n*=8, Magnets, CRY2/CIB: *n*=4, mean±s.d.).

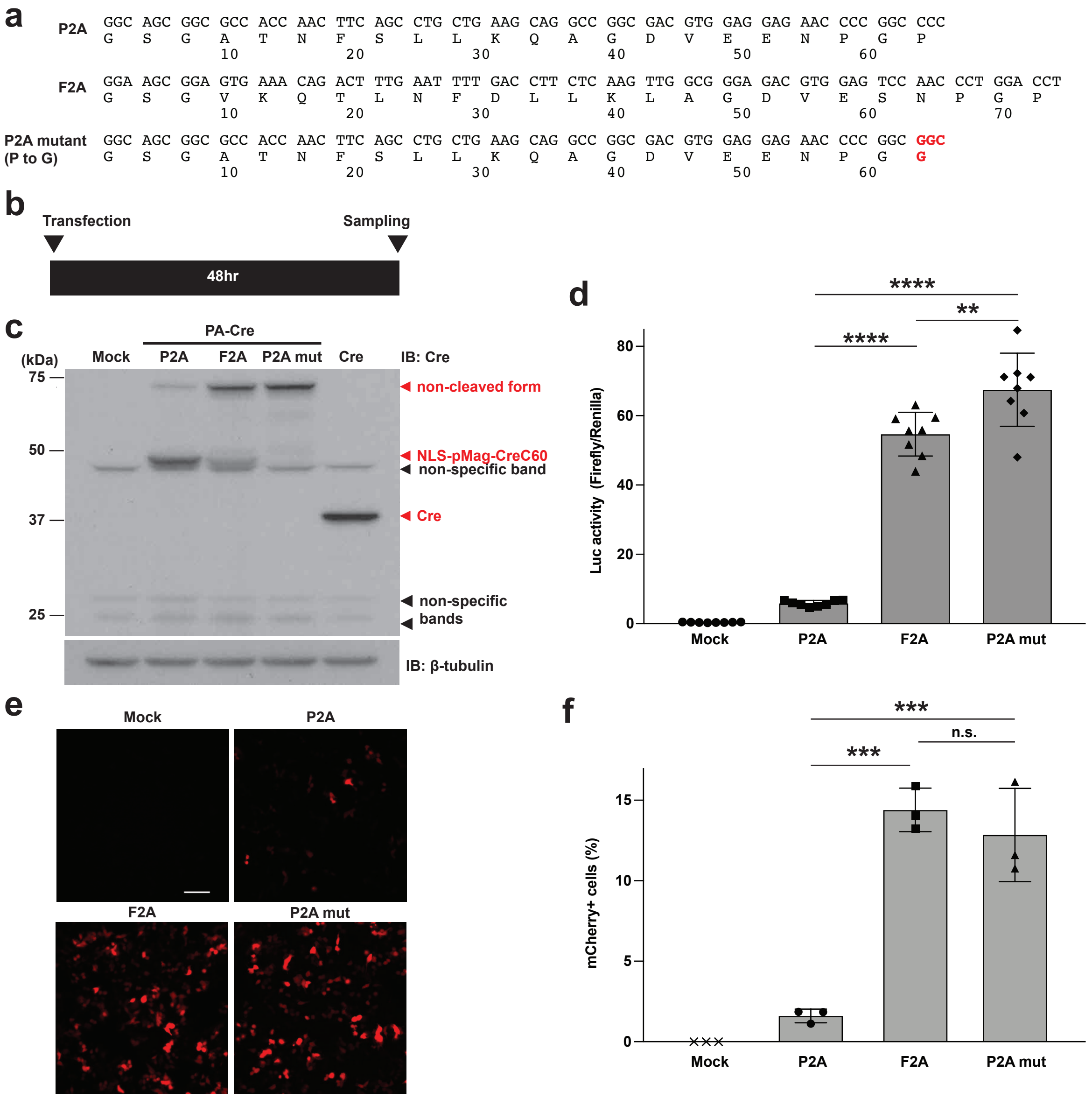

Supplementary Fig. 2 Test 2A cleaving peptide variants and comparison of PA-Cre constructs

Supplementary Fig. 2 Test 2A cleaving peptide variants and comparison of PA-Cre constructs.

a, Alignment of 2A self-cleaving peptide sequences. The sequences showed the original P2A, F2A and P2A non-cleaving mutants. The red amino acid indicated the nonfunctional mutation of P2A (P to G mutation).

b, Diagram shows the experimental time course. HEK 293T cells transfected with CMV promoter-mediated Magnets-based 2A variants and reporter plasmids transiently. The cells were incubated in the dark for 48 h.

c, Representative Western blotting using Magnets-based PA-Cre with P2A, F2A and P2A mutant (P2A mut) in HEK 293T cells in dark condition. The different 2A versions of PA-Cre proteins were detected by Cre antibody. A house keeping molecule, β-tubulin, was examined as an internal control in the cells (single independent experiments).

d, Luciferase assay for comparison of leak activities in the dark condition using Magnets-based PA-Cre with P2A, F2A and P2A mutants (**$P<0.005$, ****$P<0.0001$; one-way ANOVA with multiple comparison among P2A, F2A and P2A mutant, $n=8$ biologically independent samples, mean±s.d.).

e, Representative images of mCherry fluorescence in the cells ($n=3$ independent experiments). Scale bar, 100μm.

f, Quantification of mCherry-positive cells in the dark condition (n.s. not significant, ***$P<0.001$; one-way ANOVA with multiple comparisons, $n=3$ biologically independent samples, mean±s.d.).

Each sequences is highlighted as follows:

Cloning site (NheI/KpnI/BamHI)   Start codon   CreN59   nMag   NLS

Seq_1 (upper): pcDNA3-Magnets
Seq_2 (lower): pcDNA3-Magnets-opti

```
              NheI                            Start      CreN59
Seq_1   1     GCTAGCGTTTAAACTTAAGCTTACCACCATGGCCACCTCTGATGAAGTCAGGAAGAACCT  60
              ||||||||||||||||||||||#||||||||||#|||||#||||||||##||||#|||||
Seq_2   1     GCTAGCgtttaaacttaagcttgccaccatggctacctccgatgaagtgcggaaaaacct  60

Seq_1   61    GATGGACATGTTCAGGGACAGGCAGGCCTTCTCTGAACACACCTGGAAGATGCTCCTGTC  120
              ||||||#||||||#|#|||#|#||#|||||###|||#||#||#|||||#|||#|#||###
Seq_2   61    gatggatatgttccgcgaccgacaagcctttagtgagcatacatggaaaatgttgcttag  120

                                                   KpnI     nMag
Seq_1   121   TGTGTGCAGATCCTGGGCTGCCTGGTGCAAGCTGAACGGTACCCATACTCTTTATGCCCC  180
              #||#|||||#||#|||||#||#||||||||#||||||||#|||||#||#|||||||||||
Seq_2   121   cgtttgcaggtcatgggccgcttggtgcaaactgaacgggacccacaccctttatgcccc  180

Seq_1   181   CGGTGGATATGACATTATGGGATATCTGGACCAGATCGGCAACCGGCCAAACCCGCAGGT  240
              #|||||#||||||||||||||#|||||#|||||#||#||||||#||||#||#||#|||||
Seq_2   181   tggtggttatgacattatggggtatctcgaccaaataggcaacaggcccaatccacaggt  240

Seq_1   241   GGAACTGGGCCCCGTGGATACATCCTGCGCCTTGATTCTTTGTGACCTGAAACAGAAAGA  300
              #|||#||||#|||||#||#||#||#||||||#|#||#||#||||||#|||||||#||#||
Seq_2   241   tgaattggggcccgttgacacttcttgcgcccttatactctgtgacttgaaacaaaagga  300

Seq_1   301   CACCCCGATAGTTTACGCGAGTGAAGCCTTCCTCTACATGACAGGTTACAGCAACGCAGA  360
              #||#||#|||||#|||||#||#||#||#||#|||||#||||||||#|||##|||||||||
Seq_2   301   tacacccatagtgtacgccagcgaggcatttctctatatgacaggctactccaacgcaga  360

Seq_1   361   GGTGCTGGGCCGGAATTGCCGGTTTCTGCAAAGCCCTGACGGCATGGTGAAGCCCAAGAG  420
              |||#|||||##||||#||#||||||||#|||||#||#||#||#|||||#||#||||||##
Seq_2   361   ggtcctgggtaggaactgtcggtttcttcaaagtccagatggtatggtcaaacccaagtc  420

Seq_1   421   CACCCGGAAGTACGTGGATAGTAACACAATCAATACTATGCGCAAGGCAATCGACAGGAA  480
              |||##||||#|||||#||####||#||||||||#||#|||#|#|||||#||#||##|#||
Seq_2   421   cacaaggaaatacgtagactccaatacaatcaacacaatgaggaaggccatagatcgaaa  480

Seq_1   481   TGCCGAGGTGCAGGTTGAAGTAGTCAATTTTAAAAAGAATGGACAGCGATTTGTTAATTT  540
              #||#|||||#|||||#|||||#||#||#|||||#||#||||||||#|||||#|||||#||
Seq_2   481   cgcagaggttcaggtggaagtggttaactttaagaaaaatggacaacgattcgttaactt  540

Seq_1   541   CCTGACTATGATACCTGTTAGGGACGAAACAGGCGAGTATCGATACTCTATGGGATTCCA  600
              |#||||||||||||||||##|||||||#||#||#||||||#|#||#||#|||||#|||||
Seq_2   541   cttgactatgatacctgtgcgggacgagactggggagtataggtattcaatggggttcca  600

                                                NLS            BamHI
Seq_1   601   GTGCGAAACAGAAGGCGGAAGCGGTGGCGTGCCCAAGAAGAAGAGGAAAGTCGGATCC  658
              |||#|||||#|||||#||####|||||#||#||#||||||||##||||#|||||||||
Seq_2   601   gtgtgaaaccgaaggggggtcaggtggggtcccaaagaagaaacggaaggtcGGATCC  658
```

Supplementary Fig. 3 Sequence alignment of Magnets and Magnets-optimized constructs

Supplementary Fig. 3 Sequence alignment of Magnets and Magnets-optimized constructs.

Sequence_1 (upper sequence) is Magnets and Sequence_2 (lower sequence) is Magnets-opti. # highlighted the difference.

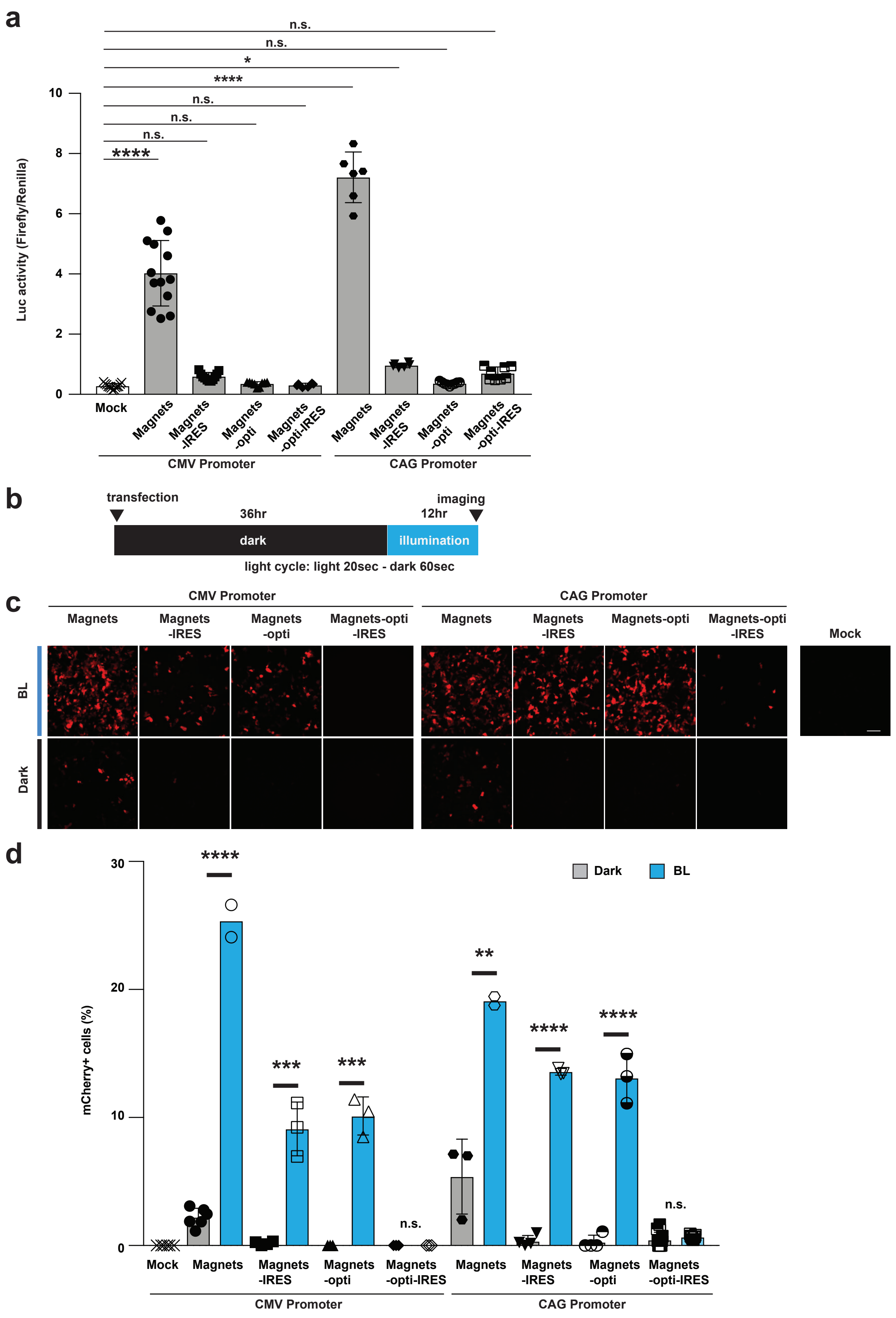

Supplementary Fig. 4 Codon modification and CAG promoter activity improve PA-Cre function.

Supplementary Fig. 4 Codon modification and CAG promoter activity improve PA-Cre function.

a, Comparison of PA-Cre leak activity in the dark. Luc assay was conducted using double-floxed inverted Fluc reporter in HEK 293T cells (same dataset shown in Fig. 2c). Transfected cells were kept in the dark for 48 h. (n.s. not significant, **P*<0.05, *****P*<0.0001; one-way ANOVA, Biologically independent samples; Mock, CMV-Magnets: *n*=13, CMV-Magnets-IRES *n*=11, CMV-Magnets-opti *n*=9, CMV-Magnets-opti-IRES, CAG-Magnets, CAG-Magnets-IRES: *n*=6, CAG-Magnets-opti *n*=9, CAG-Magnets-opti-IRES2 *n*=8, mean±s.d.).

b, Schematic representation of experimental time course (blue LED, 447.5nm, 8.28W/m^2, repeated 20-sec light and 60-sec dark cycle for 12 h).

c, Representative mCherry red fluorescence images of HEK 293T cells expressing multiple Magnets- and Magnets-opti-based constructs illuminated by blue light (BL) or kept in the dark (*n*=2 biologically independent samples, Scale bar: 100μm).

d, Quantification of mCherry-positive cells expressing the PA-Cre constructs in dark and BL conditions (n.s. not significant, ***P*<0.01, ****P*<0.0005, *****P*<0.0001; BL v.s. dark using Two-tailed *t*-test, Biologically independent samples; Mock, CMV-Magnets Dark: *n*=6, CMV-Magnets BL, CAG-Magnets BL: *n*=2, CMV-Magnets-IRES Dark, CAG-Magnets-IRES Dark, CAG-Magnets-opti Dark: *n*=4, CMV-Magnets-IRES BL, CMV-Magnets-opti Dark, CMV-Magnets-opti BL, CMV-Magnets-opti-IRES Dark, CMV-Magnets-opti-IRES BL, CAG-Magnets Dark, CAG-Magnets-IRES BL, CAG-Magnets-opti BL: *n*=3, CAG-Magnets-opti-IRES Dark *n*=10, CAG-Magnets-opti-IRES BL *n*=9, mean±s.d.).

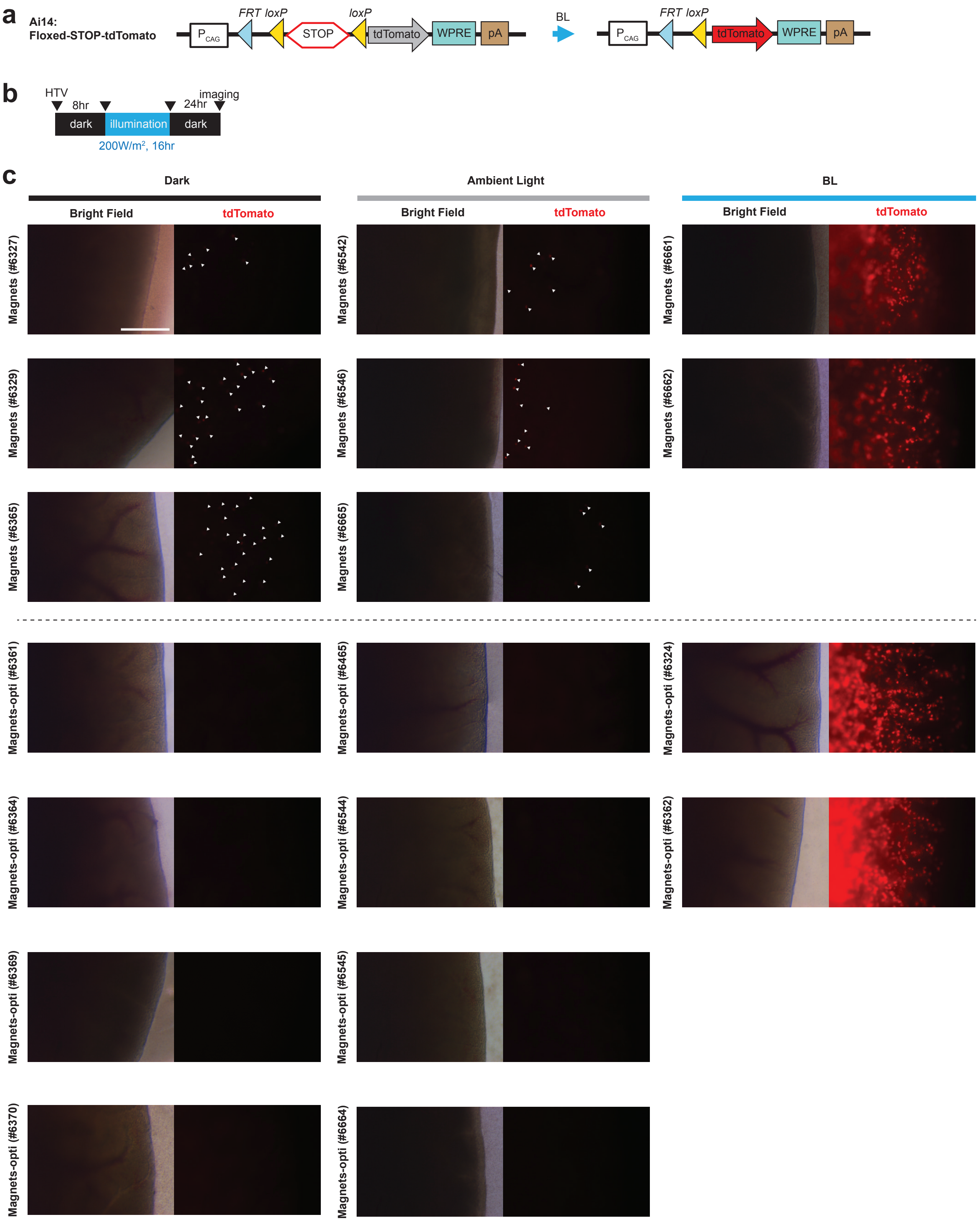

Supplementary Fig. 5 The original Magnets version shows the leak activity in the dark with multiple mice *in vivo*.

Supplementary Fig. 5 The original Magnets version shows the leak activity in the dark with multiple mice *in vivo*.

a, Schematics depicting the generation of Cre-reporter *Rosa26*$^{Ai14/WT}$ mice. Cre reporter (tdTomato) allele are located on the *Rosa26* locus in *Rosa26*$^{Ai14/WT}$ mouse line. PA-Cre-mediated excision of the *loxP*-flanked stop cassette (Stop) controlled by blue light illumination induces the expression of tdTomato red fluorescence protein.

b, Schematic representation of experimental time course.

c, Fluorescence images of livers isolated from multiple mice. tdTomato red fluorescent protein expression was accomplished in *Rosa26*$^{Ai14/WT}$ living mice transiently injected with CAG promoter-mediated Magnets (original) or CAG promoter-mediated Magnets-optimized version (Magnets-opti) plasmids, respectively in dark, ambient light or under blue light illumination (470±20nm, 200W/m^2, 16 h continuous). White arrowheads show tdTomato-positive liver cells (Scale bar: 1mm, *n*=2-6 mice/group). The mouse ID # were also shown in the experimental groups.

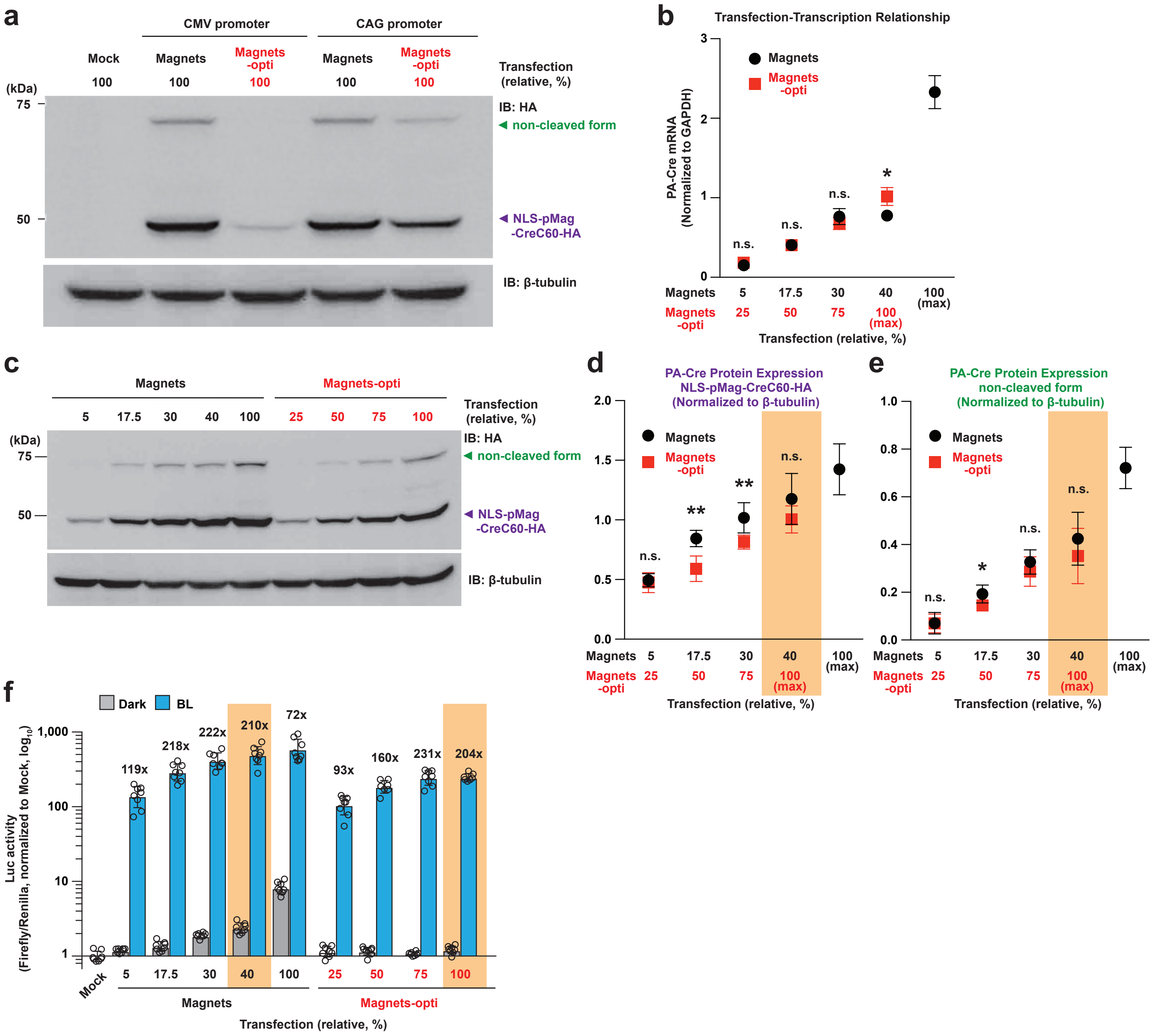

Supplementary Fig. 6 Comparison of PA-Cre constructs using various transfection conditions

Supplementary Fig. 6 Comparison of PA-Cre constructs using various transfection conditions.

a, Representative Western blotting HEK 293T cells transfected with CMV-Magnets-HA, CMV-Magnets-opti-HA, CAG-Magnets-HA and CAG-Magnets-opti-HA kept in dark. PA-Cre proteins were detected by HA antibody. Top band (~75kDa) shows non-cleaved form of PA-Cre and bottom band shows cleaved c-term portion of PA-Cre (NLS-pMag-CreC60-HA). β-tubulin, was examined as an internal control (*n*=2).

b, Quantification of PA-Cre transcripts in HEK 293T cells transfected with CAG-Magnets-HA or CAG-Magnets-opti-HA plasmids with multiple plasmid DNA amount. The X-axis indicates relative transfection plasmid amounts of CAG-Magnets-HA (black) and CAG-Magnets-opti-HA (red). Empty vector was used to adjust total transfection. The Y-axis shows PA-Cre transcripts, which were normalized to GAPDH (n.s. not significant, *P<0.05; Magnets v.s. Magnets-opti using Two-tailed t-test, biologically independent samples; Magnets 5, Magnets 30, Magnets 40: n=4, others: n=6, mean±s.d.).

c, Representative Western blotting images of HEK 293T cell lysate with CAG-Magnets-HA and CAG-Magnets-opti-HA plasmids using different plasmid DNA amounts. The loading schematics show relative transfection plasmid amounts of CAG-Magnets-HA or CAG-Magnets-opti-HA. HA-tagging PA-Cre was detected by HA antibody. All protein expressions were kept in the dark. β-tubulin was examined as an internal control (n=6 biologically independent samples).

d, Quantification of NLS-pMag-CreC60-HA cleaved component of PA-Cre constructs. The X-axis shows relative transfection plasmid amounts of CAG-Magnets-HA (black) and CAG-Magnets-opti-HA (red). The Y-axis shows PA-Cre cleaved form proteins, which were normalized to β-tubulin (n.s. not significant, **P<0.005; Magnets v.s. Magnets-opti using Two-tailed t-test, n=6 biologically independent samples, mean±s.d.).

e, Quantification of non-cleaved form of PA-Cre constructs. The X-axis shows relative transfection plasmid amounts of CAG-Magnets-HA (black) and CAG-Magnets-opti-HA (red). The Y-axis shows PA-Cre non-cleaved proteins, which were normalized to β-tubulin (n.s. not significant, *P<0.05; Magnets v.s. Magnets-opti using Two-tailed t-test, n=6 biologically independent samples, mean±s.d.).

f, Comparison of PA-Cre recombinase activities between Magnets and Magnets-opti without and with illumination. The X-axis shows relative transfection plasmid amounts of CAG-Magnets-HA (black) and CAG-Magnets-opti-HA (red). The Y-axis shows luciferase activity (Firefly normalized to Renilla, a transfection control), which were also normalized to Mock. Transfected cells were kept in the dark or under blue light. The black numbers show BL-mediated Luc fold-induction value (Biologically independent samples; Magnets-opti 50 BL, Magnets-opti 100 BL: n=7, others: n=8, mean±s.d.).

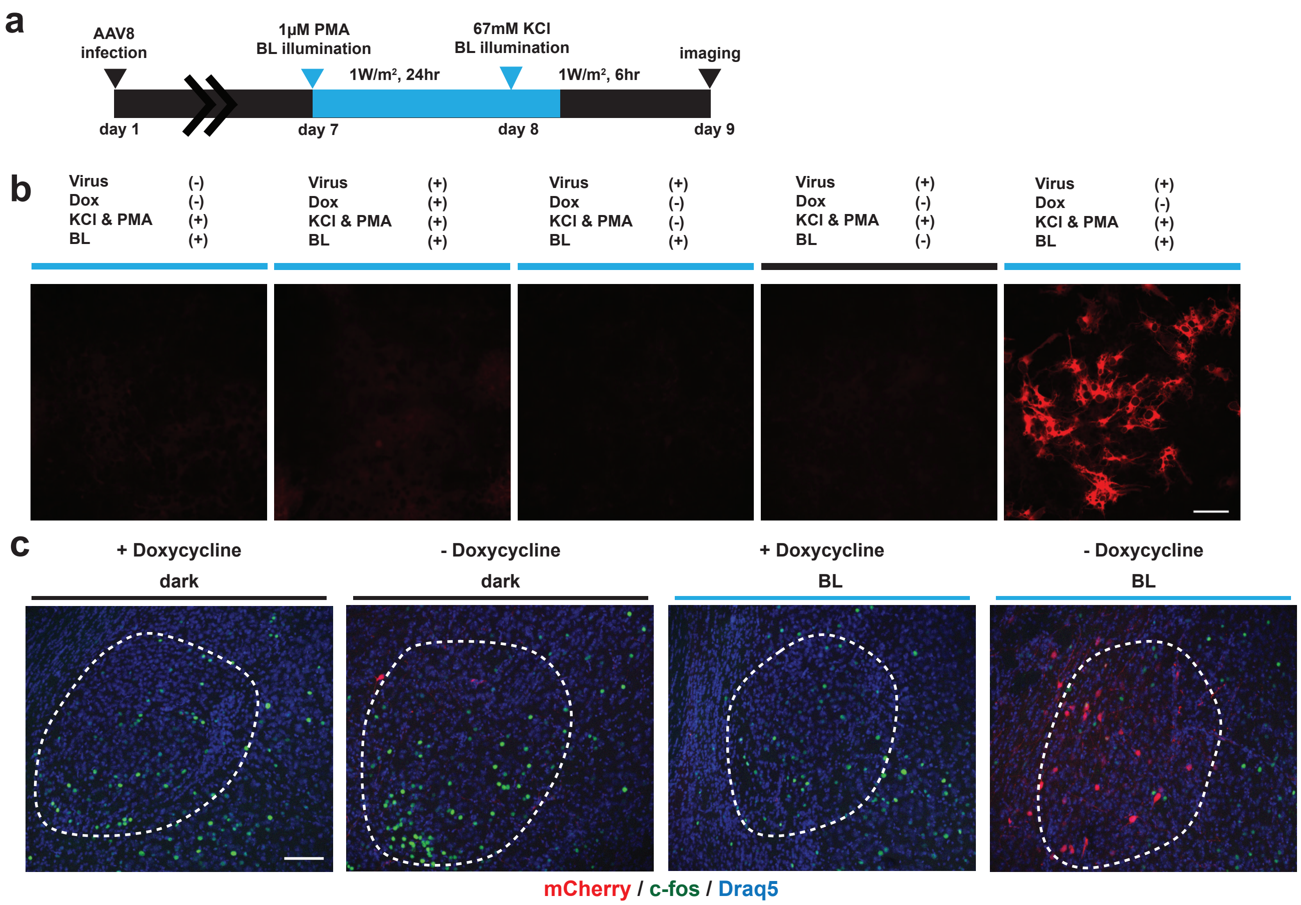

Supplementary Fig. 7 Application of AAV-RAM-Tet-off-PA-Cre 3.0 in primary neuronal cells and mousen brains

Supplementary Fig. 7 Application of AAV-RAM-Tet-off-PA-Cre 3.0 in primary neuronal cells and mouse brains.

a, Schematic representation of experimental time course. The neural progenitor cells (NPCs) isolated from *Rosa26*$^{Ai14/WT}$ E12.5 embryos and were differentiated into cortical neurons with brain-derived neurotrophic factor (BDNF) and neurotrophin-3 (NT3) treatment. To increase the *c-fos* activity, PMA (12-myristate-13-acetate) was treated with 24 h blue light illumination 1 week after AAV-RAM-Tet-off-PA-Cre3.0 infection. Following the PMA treatment, KCl was also treated with 6 h blue light illumination to increase *c-fos* activity. The tdTomato red fluorescence was detected at the following day after the blue light illumination (470±20nm, 1W/m^2, continuous).

b, Representative tdTomato red fluorescence images in AAV-RAM-Tet-off-PA-Cre3.0-infected NPC-derived neurons in various conditions using PMA, Dox, KCl and BL (Scale bar: 100µm, *n*=2-3 independent experiments).

c, Representative immunofluorescent images of red fluorescent protein, mCherry (red) and neuronal activity marker, c-fos (green) and Draq5 (blue, nuclear staining) in amygdala (dashed line) infected with AAV8-RAM-Tet-off-PA-Cre 3.0 and AAV8-hSyn-DIO-mCherry viruses in doxycycline diet-fed (+ Doxycycline) and regular diet (- Doxycycline) groups in the conditions of dark and BL illumination (each, *n*=3-4). Scale bar, 100µm.

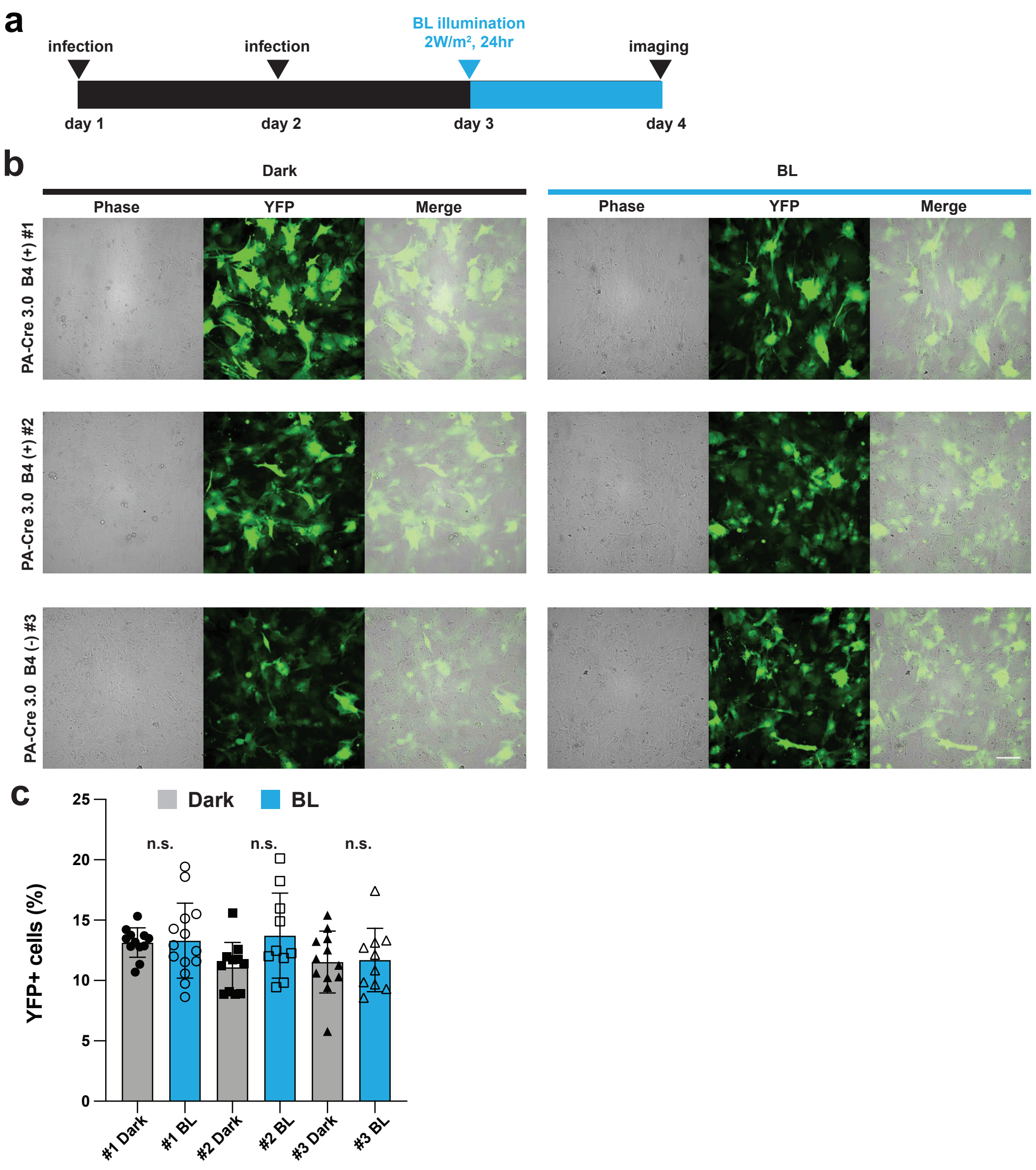

Supplementary Fig. 8 Infection control experiments in PA-Cre MEFs using YFP viruses.

Supplementary Fig. 8 Infection control experiments in PA-Cre MEFs using YFP viruses.

a, Schematic representation of experimental time course.

b, Representative yellow fluorescent protein (YFP) fluorescence images in lentiviral YFP-infected PA-Cre 3.0 MEFs (Scale bar: 100μm, *n*=2 independent experiments).

c, Quantification of YFP-positive MEFs in dark and BL conditions (n.s. not significant; one-way ANOVA with multiple comparison, Biologically independent samples; #1 Dark *n*=12, #1 BL *n*=14, #2 Dark *n*=11, #2 BL, #3 BL: *n*=10, #3 Dark *n*=12, mean±s.d.).

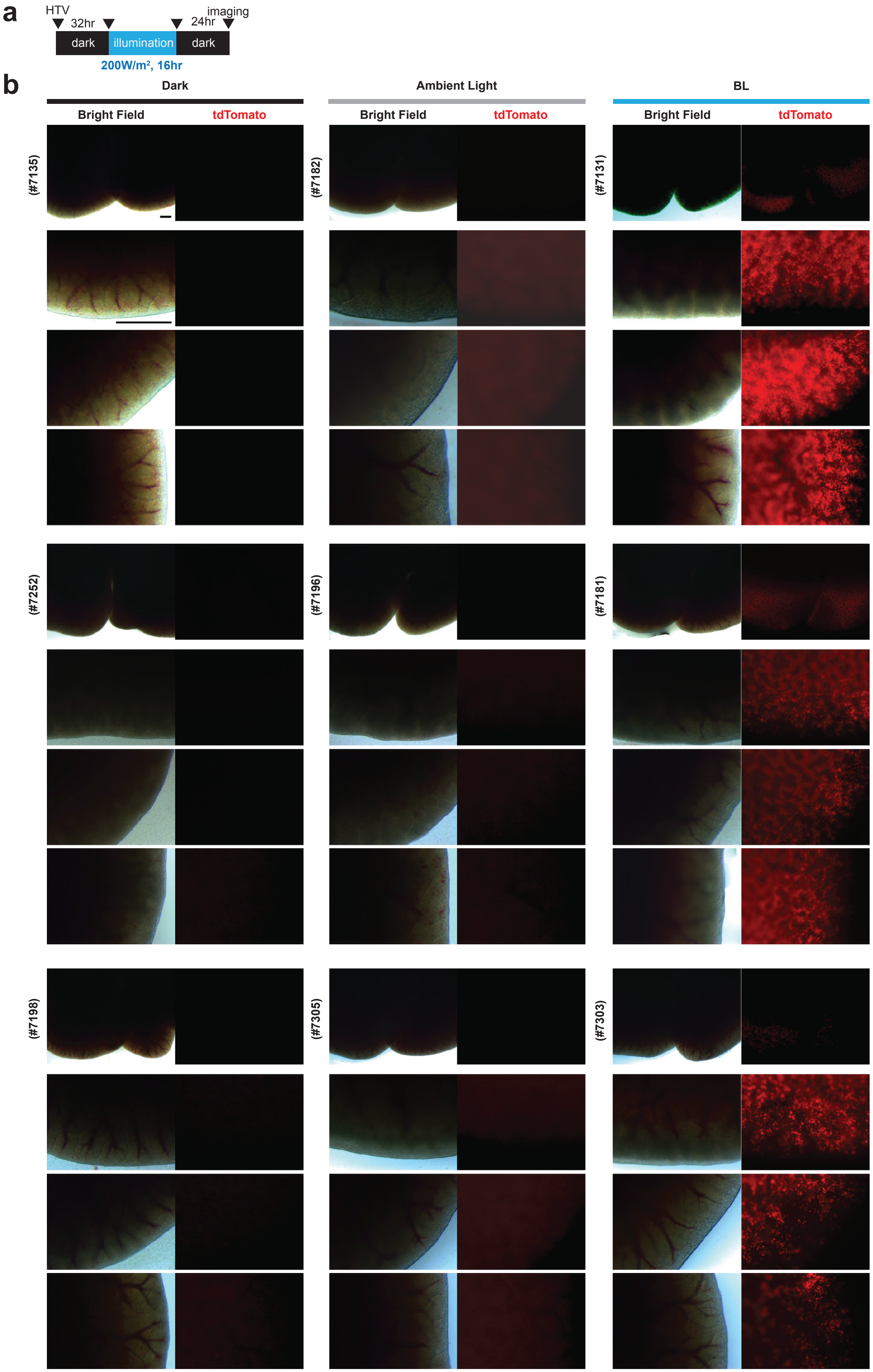

Supplementary Fig. 9 *in vivo* **single genome recombination in ROSA26$^{PA\text{-}Cre\ B4/Ai14}$ mice**

Supplementary Fig. 9 *in vivo* single genome recombination in *Rosa26*$^{PA\text{-}Cre\ B4/Ai14}$ mice.

a, Schematic representation of experimental time course. After hydrodynamic injection of CAG-Flpe plasmid, the mice were maintained in the dark, ambient light or blue light illumination (BL) (470±20nm, 200W/m^2, 16 h continuous). Seventy-two hours after initial injection, the mice were sacrificed to obtain their liver fluorescence images.

b, Representative fluorescence images of liver tissues isolated from *Rosa26*$^{PACre\ B4/Ai14}$ mice injected with CAG-Flpe plasmids. Upper images show the whole livers in low magnification (Scale bar: 1mm). Lower images indicate multiple liver locations in higher magnification from each mouse (Scale bar: 0.5mm) (*n*=3-4 mice/group).

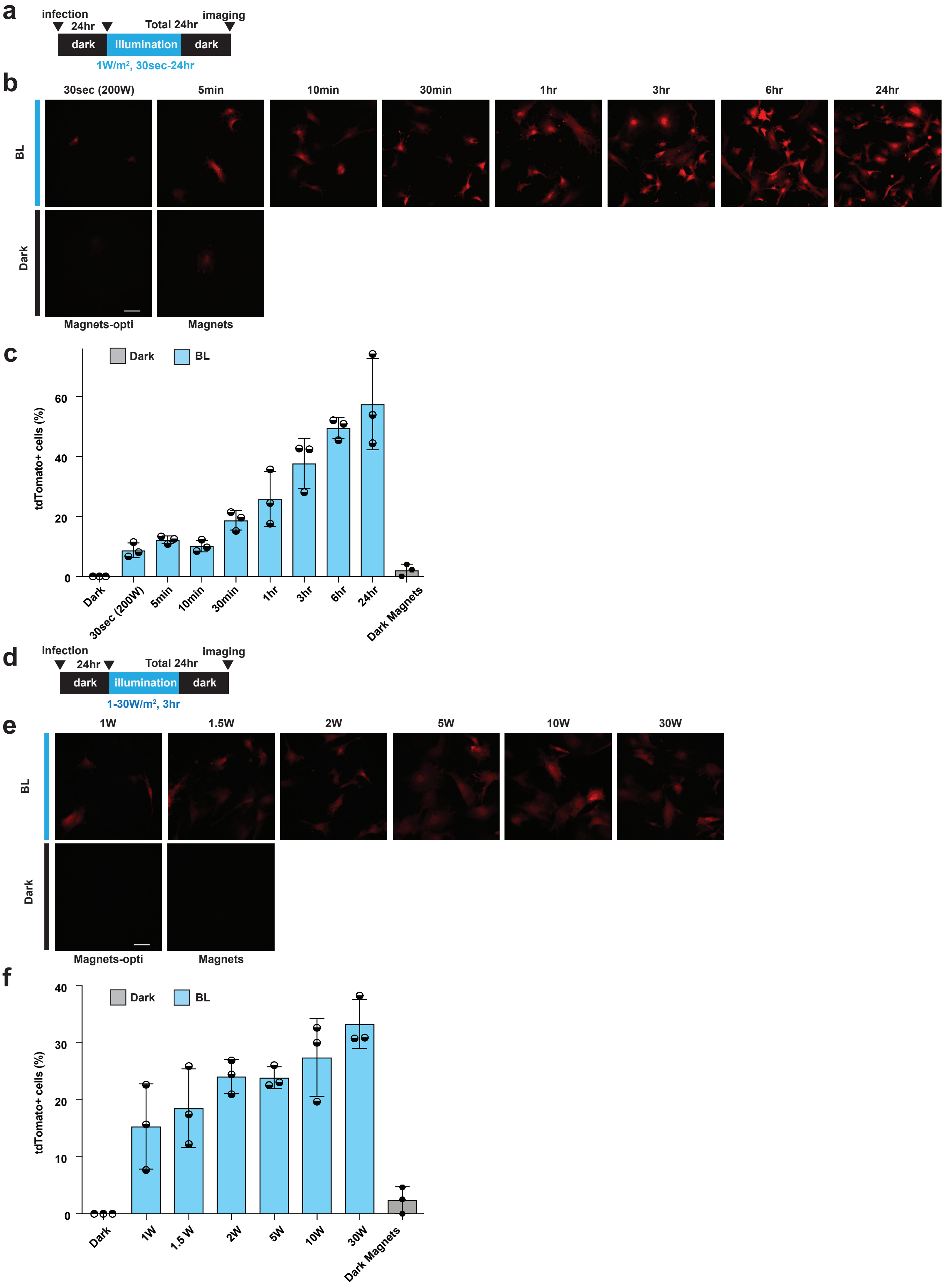

Supplementary Fig. 10 Single target of loxP pair in the genome of mouse embryonic fibroblasts by using PA-Cre 3.0 lentivirus

Supplementary Fig. 10 Single target of loxP pair in the genome of mouse embryonic fibroblasts by using PA-Cre 3.0 lentivirus.

a, Schematic representation of experimental time course. The mouse embryonic fibroblasts (MEFs) isolated from *Rosa26*$^{Ai14/WT}$ E13.5 embryos. Twenty-four hours after CAG promoter-mediated Magnets-opti PA-Cre (PA-Cre 3.0) lentiviral infection, the reporter MEFs were illuminated with various periods of blue light (470±20nm, 1W/m^2/5min-24h continuous, 200W/m^2/30sec). The tdTomato fluorescence images were observed 24h after a beginning of illumination.

b, Representative red fluorescent images of tdTomato in the MEFs with various illumination protocols (*n*=3 independent experiments, Scale bar: 100µm).

c, Comparison of PA-Cre 3.0 responses in various periods of blue light illumination (*n*=3 independent experiments, mean±s.d.).

d, Schematic representation of experimental time course. Twenty-four hours after CAG promoter-mediated Magnets-opti PA-Cre lentiviral infection, the *Rosa26*$^{Ai14/WT}$ MEFs were illuminated with various power of blue light for 3 h (70±20nm, 1-30W/m^2, 3 h continuous). The tdTomato red fluorescence images were captured 24 h after the illumination started.

e, Representative images of tdTomato in the MEF cells under different light intensities (*n*=3 independent experiments, Scale bar, 100µm).

f, Light power dependence of PA-Cre 3.0 with 3h illumination (*n*=3 independent experiments, mean±s.d.).
