# Supplementary material for: Photoactivatable Cre recombinase 3.0 for in vivo mouse applications
Source: Nat Commun. 2020 May 1;11:2141. doi: 10.1038/s41467-020-16030-0 (PMC7195411; doi:10.1038/s41467-020-16030-0)
Supplement: Supplementary file 3 — Reporting Summary [file 41467_2020_16030_MOESM3_ESM.pdf]

nature research

Corresponding author(s): Masayuki Yazawa

Last updated by author(s): Mar 20, 2020

Reporting Summary

Nature Research wishes to improve the reproducibility of the work that we publish. This form provides structure for consistency and transparency in reporting. For further information on Nature Research policies, see Authors & Referees and the Editorial Policy Checklist.

Statistics

For all statistical analyses, confirm that the following items are present in the figure legend, table legend, main text, or Methods section.

n/a	Confirmed	
☐	☒	The exact sample size (*n*) for each experimental group/condition, given as a discrete number and unit of measurement
☐	☒	A statement on whether measurements were taken from distinct samples or whether the same sample was measured repeatedly
☐	☒	The statistical test(s) used AND whether they are one- or two-sided<br>*Only common tests should be described solely by name; describe more complex techniques in the Methods section.*
☐	☒	A description of all covariates tested
☐	☒	A description of any assumptions or corrections, such as tests of normality and adjustment for multiple comparisons
☐	☒	A full description of the statistical parameters including central tendency (e.g. means) or other basic estimates (e.g. regression coefficient) AND variation (e.g. standard deviation) or associated estimates of uncertainty (e.g. confidence intervals)
☐	☒	For null hypothesis testing, the test statistic (e.g. *F*, *t*, *r*) with confidence intervals, effect sizes, degrees of freedom and *P* value noted<br>*Give P values as exact values whenever suitable.*
☒	☐	For Bayesian analysis, information on the choice of priors and Markov chain Monte Carlo settings
☒	☐	For hierarchical and complex designs, identification of the appropriate level for tests and full reporting of outcomes
☒	☐	Estimates of effect sizes (e.g. Cohen's *d*, Pearson's *r*), indicating how they were calculated

Our web collection on statistics for biologists contains articles on many of the points above.

Software and code

Policy information about availability of computer code

Data collection	GLOMAX-Version 1.9.2 (Promega), NIS Elements Version 4.51.01 (Nikon), Axio Vision Release 4.8.2 (Carl Zeiss), StepOne Software Version 2.3 (Life Technologies), LIF
Data analysis	Microsoft Excel for Mac 2011 (Microsoft), Prism Version 7.0d (Graphpad), NIS Elements Version 5.02.00 (Nikon), Serial Cloner Version 2.6.1, ImageJ-win64 (Fiji), LAS X (Leica Microsystems)

For manuscripts utilizing custom algorithms or software that are central to the research but not yet described in published literature, software must be made available to editors/reviewers. We strongly encourage code deposition in a community repository (e.g. GitHub). See the Nature Research guidelines for submitting code & software for further information.

Data

Policy information about availability of data

All manuscripts must include a data availability statement. This statement should provide the following information, where applicable:
- Accession codes, unique identifiers, or web links for publicly available datasets
- A list of figures that have associated raw data
- A description of any restrictions on data availability

Data available on request from the authors.

Field-specific reporting

Please select the one below that is the best fit for your research. If you are not sure, read the appropriate sections before making your selection.

☒ Life sciences ☐ Behavioural & social sciences ☐ Ecological, evolutionary & environmental sciences

Life sciences study design

All studies must disclose on these points even when the disclosure is negative.

Sample size	Power studies were conducted to minimize animal use following our IACUC protocol.
Data exclusions	Individual data points were excluded based on the Grubb's outlier statistical analysis.
Replication	Experiments were conducted in at least two independent experiments (e.g. two separately prepared experiments)
Randomization	N/A. There was no need for randomization in the study
Blinding	N/A. There was no need for blinding in this study

Reporting for specific materials, systems and methods

We require information from authors about some types of materials, experimental systems and methods used in many studies. Here, indicate whether each material, system or method listed is relevant to your study. If you are not sure if a list item applies to your research, read the appropriate section before selecting a response.

Materials & experimental systems

n/a	Involved in the study	
☐	☒	Antibodies
☐	☒	Eukaryotic cell lines
☒	☐	Palaeontology
☐	☒	Animals and other organisms
☒	☐	Human research participants
☒	☐	Clinical data

Methods

n/a	Involved in the study	
☒	☐	ChIP-seq
☒	☐	Flow cytometry
☒	☐	MRI-based neuroimaging

Antibodies

Antibodies used	Cre (Abcam, #ab24608), HA (Roche, #11867423001), β-tubulin (Sigma-Aldrich, #t5201), c-fos (Calbiochem, ABE457), c-fos (Synaptic Systems, #226003), mCherry (Invitrogen, #16D7), HRP conjugate α-mouse (Thermo Scientific, #31430), HRP conjugate α-rabbit (Thermo Scientific, #31460), HRP conjugate α-rat (Thermo Scientific, #31470), Biotinylated donkey anti-rabbit (Jackson ImmunoResearch), Avydin-Cy3 (Jackson ImmunoResearch), Alexa 488-goat α-rabbit (Thermo Scientific, #A11008), Alexa 568-goat α-rat (Thermo Scientific, #A11077)
Validation	Cre Abcam antibodies were compared with other Cre antibody sources and was found to be the most specific. But Cre Abcam antibody is not commercially available. Other antibodies are widely used and cited by other publications.

Eukaryotic cell lines

Policy information about cell lines

Cell line source(s)	Human embryonic kidney (HEK) 293T cells (ATCC, #CRL-3216), Mouse embryonic stem cells
Authentication	HEK293T cells were purchased directly from ATCC. Mouse embryonic stem cells were obtained from Chyuan-Sheng Lin (Columbia Transgenic mouse facility)
Mycoplasma contamination	Mycoplasma contamination assays were completed for all the cell lines.
Commonly misidentified lines (See ICLAC register)	*Name any commonly misidentified cell lines used in the study and provide a rationale for their use.*

Animals and other organisms

Policy information about studies involving animals; ARRIVE guidelines recommended for reporting animal research

Laboratory animals	Mus musculus, Male and Female. Strain: C57Bl6/J, B6.Cg-Gt(rOSA)26Sortm14(CAG-tdTomato)Hze/J; (The Jackson Laboratory, JAX# 007914), B6.129S4-Gt(ROSA)26Sortm1(FLP1)Dym/RainJ (The Jackson Laboratory, JAX# 009086). We established novel PA-Cre chimeric mice in Columbia Transgenic mouse facility. The detailed informations are specifically described in "Animals" section of the Methods.

Wild animals	Study does not involve wild animals.
Field-collected samples	Study does not involve samples collected from the field.
Ethics oversight	Institutional Animal Care and Use Committee at Columbia University (#AC-AAAU2453) and Louisiana State University Health Sciences Center (#3695) approved all animal experiments

Note that full information on the approval of the study protocol must also be provided in the manuscript.
