# Supplementary material for: Photoactivatable Cre recombinase 3.0 for in vivo mouse applications
Source: Nat Commun. 2020 May 1;11:2141. doi: 10.1038/s41467-020-16030-0 (PMC7195411; doi:10.1038/s41467-020-16030-0)

Western Blotting Source: Film Images

Fig. 2b anti-Cre blotting

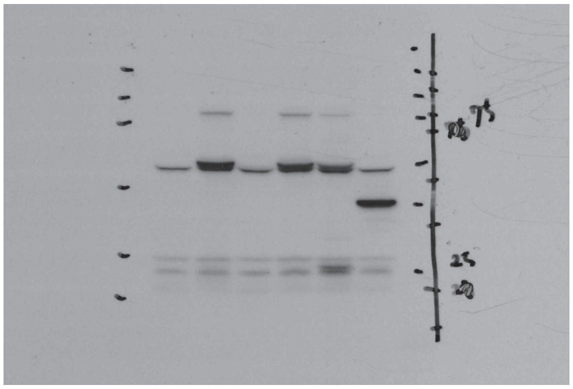

Fig. 2b anti-β-tubulin blotting

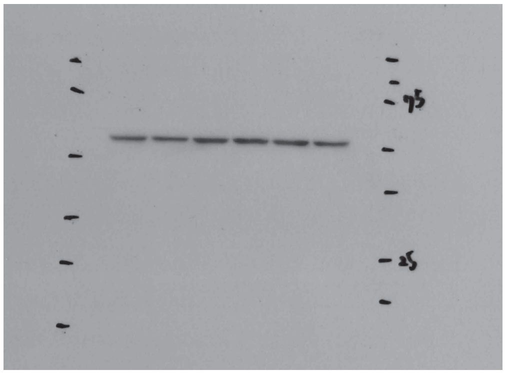

Fig. 3d anti-HA blotting

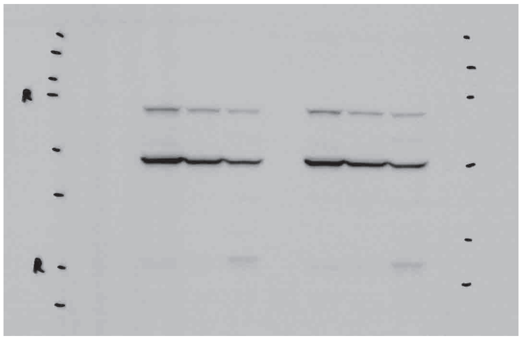

Fig. 3d anti-β-tubulin blotting

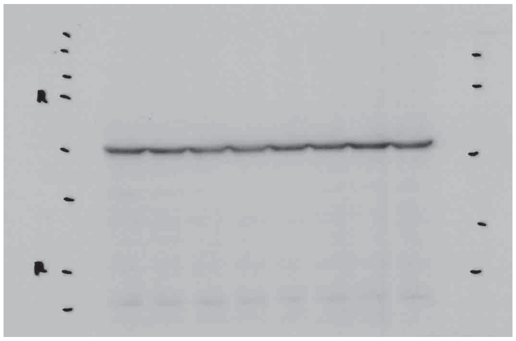

Sup Fig. 2c anti-Cre blotting

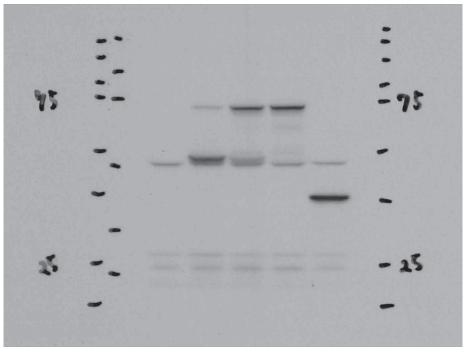

Sup Fig. 2c anti-β-tubulin blotting

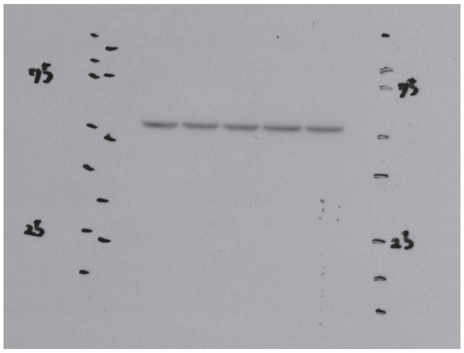

Sup Fig. 6a anti-HA blotting

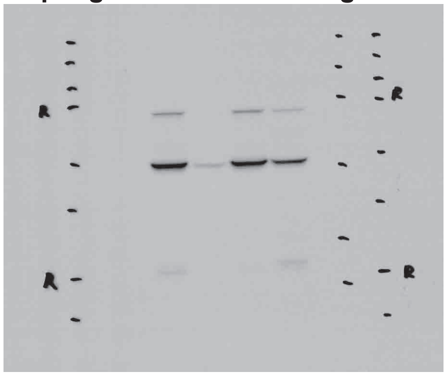

Sup Fig. 6a anti-β-tubulin blotting

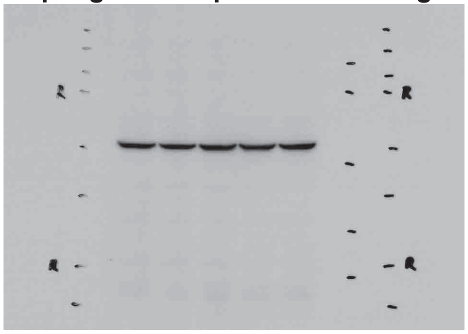

Sup Fig. 6c anti-HA blotting

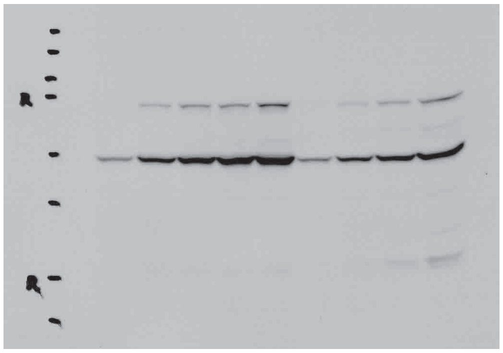

Sup Fig. 6c anti-β-tubulin blotting

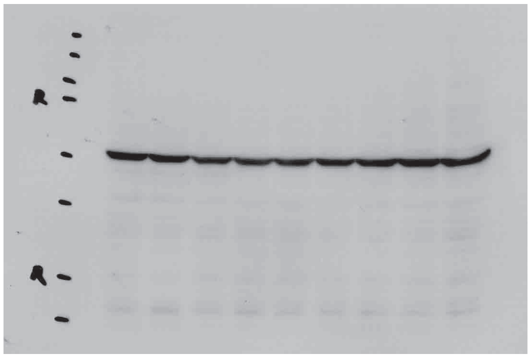

Supplement: Supplementary file 4 — Source Data [file 41467_2020_16030_MOESM4_ESM.zip › Source Data - Western Blotting Film Images.pdf]
